# Tomato-Derived Lycopene: From Phytochemistry and Extraction Technologies to Bioavailability and Nutraceutical Applications

**DOI:** 10.3390/molecules31132243

**Published:** 2026-06-25

**Authors:** Andra-Monica Anghel (Ştefan), Elena Enachi, Alina-Georgiana Cristea (Hohotă), Fănică Bălănescu, Oana Cioancă, Monica Hăncianu, Silvia Robu

**Affiliations:** 1 Research Centre in the Medical-Pharmaceutical Field, Faculty of Medicine and Pharmacy, “Dunărea de Jos” University, 800008 Galati, Romaniafanica.balanescu@ugal.ro (F.B.); silvia.robu@ugal.ro (S.R.); 2 Faculty of Pharmacy, “Grigore T. Popa” University of Medicine and Pharmacy, 16 Universitatii Street, 700115 Iasi, Romania; oana.cioanca@gmail.com (O.C.); monica.hancianu@gmail.com (M.H.)

**Keywords:** tomatoes, phytochemicals, lycopene, dietary intake vs. supplement intake, biological activities

## Abstract

Tomatoes (*Solanum lycopersicum* L.) are one of the most important dietary sources of carotenoids, especially lycopene, a bioactive compound associated with antioxidant, anti-inflammatory and cardioprotective effects. This review synthesizes recent data on the phytochemical composition of tomatoes, with a focus on lycopene, its main biological mechanisms and health benefits, including the reduction in oxidative stress. The manuscript also highlights the influence of thermal processing and food matrix on the bioavailability of lycopene, as well as the role of innovative formulation and nanoencapsulation systems in increasing its stability and absorption. Modern extraction and analysis methods are also presented, including ultrasound, microwave and supercritical-fluid-assisted techniques, along with HPLC chromatographic methods. A distinctive element is the analysis of lycopene-based food supplements available on the markets in Romania, Europe and the United States, from the perspective of composition, standardization and safety. Current data support the potential of lycopene as a valuable nutraceutical ingredient, but further clinical studies are needed to confirm therapeutic benefits.

## 1. Introduction

Tomato is one of the most important vegetables both economically and in terms of consumption [1], renowned for its versatility and dense nutrient profile. According to the Food and Agriculture Organization of the United Nations [2], tomatoes were the most produced vegetable worldwide, reaching approximately 192 million tonnes in 2023, supported by steady demand from both consumers and the industrial sector. Tomato (*Solanum lycopersicum* L.) is native to South America and belongs to the *Solanaceae* family [3], and it can be eaten fresh, dried, cooked, and processed as juice, sauce, ketchup, paste, puree, canned fruit, etc. [1]. The popularity of tomatoes is due to their health benefits and their nutrients, vitamins, mineral elements, and phytochemicals (saponins, alkaloids, flavonoids, tannins, glycosides, phenols, carotenoids—especially lycopene) [4]. Tomatoes and their derived products represent one of the primary food sources of carotenoids, providing an estimated 80% of daily intake of lycopene [5,6].

The major class of phytochemicals present in tomatoes is carotenoids, a group of compounds produced by plants and microorganisms as secondary metabolites, known for their yellow, orange, and red tones [7]. Lycopene, the dominant red pigment in ripe tomatoes, represents 60–90% of the total carotenoids in tomatoes, being the most abundant, followed by phytoene, β-carotene, and lutein [8]. Lycopene has also been found in watermelon at levels of 2.3 to 7.2 mg/100 g fresh weight (FW), and in pink grapefruit at 0.35 to 3.36 mg/100 g FW [9], while in fresh tomatoes, the concentration varies widely depending on the variety, with high-lycopene cultivars reaching approximately 14 mg/100 g FW [10]. The pigment exhibits potent antioxidant activity and has been linked to reduced oxidative stress and a lower risk of chronic diseases, such as cardiovascular disease and certain cancers. Meta-analysis studies showed that increased consumption or higher serum levels of lycopene are correlated with important cardiovascular benefits, such as reduced blood pressure, improved lipid profiles, and decreased risk of stroke [11].

Tomatoes also contain significant levels of polyphenols namely flavonoids like quercetin and naringenin, plus hydroxycinnamic acids, which synergize with carotenoids to enhance their antioxidant and anti-inflammatory activities [12].

The phytochemical composition of tomatoes shows considerable genotypic variation, with the investigated hybrids exhibiting significant differences in the levels of carotenoids, polyphenols, and vitamin C. Such variability highlights the importance of genetic background in assessing the nutritional and functional quality of tomato fruits [13].

In terms of extraction, green methods, such as ultrasound-assisted extraction (UAE) coupled with natural deep eutectic solvents (NADESs), have shown remarkable efficiency for recovering valuable bioactive compounds from tomato by-products. Other sustainable extraction approaches, including microwave-assisted extraction (MAE), enzyme-assisted extraction (EAE), pulsed electric field (PEF)-assisted extraction, pressurized liquid extraction (PLE), and supercritical fluid extraction (SFE), have also attracted increasing interest due to their reduced solvent consumption, shorter extraction times, and lower environmental impact [14]. The study by Badea et al. [15] aimed to optimize a sustainable extraction process for carotenoids and tocopherol and successfully achieved the simultaneous recovery for lycopene (215 µg/g), β-carotene (207 µg/g), and tocopherol (131 µg/g) from dried tomato by-products, with a high antioxidant capacity (93.8 mM Trolox equivalents).

Further advances have highlighted the nanostructured systems as an effective strategy to increase lycopene bioavailability. In particular, nanoemulsions and other lipid-based carriers have proven superior to traditional formulations, facilitating better dispersion and increased intestinal absorption [16].

These findings highlight the importance of tomatoes as widely consumed foods that also provide bioactive compounds with potential preventive and therapeutic relevance. This review provides a comprehensive analysis of the phytochemical potential and therapeutic relevance of *Solanum lycopersicum* (tomato) bioactive compounds, namely carotenoids, with a strong emphasis on their health-promoting properties. Carotenoids such as lycopene and beta-carotene, along with polyphenols, exhibit high antioxidant capacity, being involved in the protection of the cardiovascular system, modulation of inflammatory processes, and reduce the risk of certain types of cancer, aspects supported by experimental and clinical data. The article primarily focuses on studies published in the last decade, with particular emphasis on recent advances reported in the past 5 years and integrates the latest information on modern methods of extraction and analysis of bioactive compounds, including advanced techniques such as HPLC and mass spectrometry, as well as sustainable approaches such as ultrasound or microwave-assisted extraction, which allow for the obtaining of efficient and reproducible extracts. In addition, unlike most existing reviews, the original contribution of this study consists in the regional perspective on tomato-based supplements available on the Romanian, European and US markets, through an integrated analysis of their composition, regulation and standardization, correlated with the critical assessment of current limitations—especially regarding the translation of experimental results into clinical trials—an approach that, unlike most existing reviews, goes beyond the fragmented presentation of phytochemical composition, biological effects or extraction technologies and offers a coherent perspective with nutraceutical relevance.

## 2. Chemical Composition of Tomatoes

Environmental factors (e.g., water stress and maturity stage) as well as food processing have a major impact on the polyphenolic content. Studies show that the application of controlled water stress can lead to a significant increase in flavonoid concentration, up to 45%, thus enhancing the overall antioxidant potential of tomato-derived foods [17]. Dietary intake of polyphenols and carotenoids, especially from tomato-derived sources, is associated with protective effects against chronic diseases, acting through multiple mechanisms involving the reduction in oxidative stress, modulation of the inflammatory response, and improvement of vascular function [18].

Thermal processing (e.g., sauces, purees) may disrupt cellular matrices and enhance the release of these molecules, and co-ingestion with dietary fats, in an emulsified form, may facilitate the micellization and absorption. However, whether processed tomato products are as nutritious as fresh ones depends on multiple factors. Consequently, some processed tomato products may offer greater bioaccessible fractions than the raw fruit, depending on the processing method and formulation [8].

Besides carotenoids, it is important to note that also polyphenols and vitamins act synergistically within the tomato matrix. Flavonoids such as quercetin, rutin, and naringenin chalcone occur mainly in the peel, while phenolic acids (e.g., chlorogenic, caffeic and ferulic acids) provide an additional contribution to the overall antioxidant capacity of tomatoes [12].

### 2.1. Carotenoids

**Figure 1:**
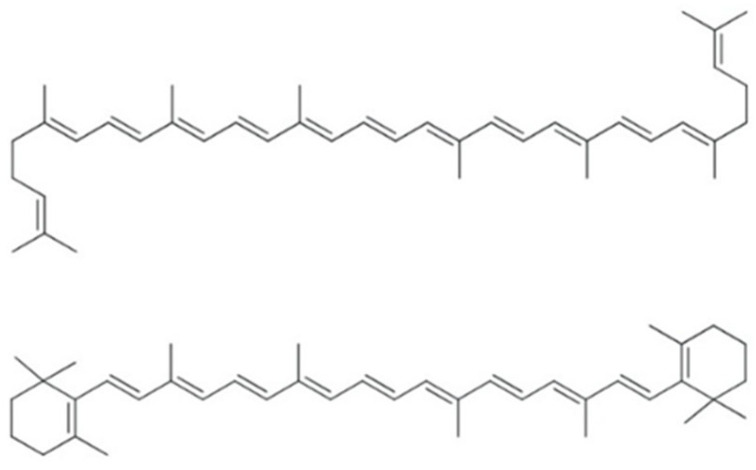
Lycopene chemical structure.

Carotenoids are a large and structurally varied family of more than 600 naturally occurring pigments synthesized by plants, algae, and other photosynthetic microbes. The sources produce the yellow, orange, and red hues of many fruits and vegetables and are crucial for plant photosynthesis/photoprotection while also supporting human health [19,20]. Although the human diet includes around 40–50 different carotenoids, only a few—such as β-carotene, α-carotene, lycopene, lutein, zeaxanthin, and β-cryptoxanthin—are typically found at measurable levels in human blood and tissues [21,22]. Among these carotenoids, lycopene is of particular interest due to its unique acyclic structure and strong antioxidant activity. The chemical structure of lycopene is presented in Figure 1.

**Figure 2:**
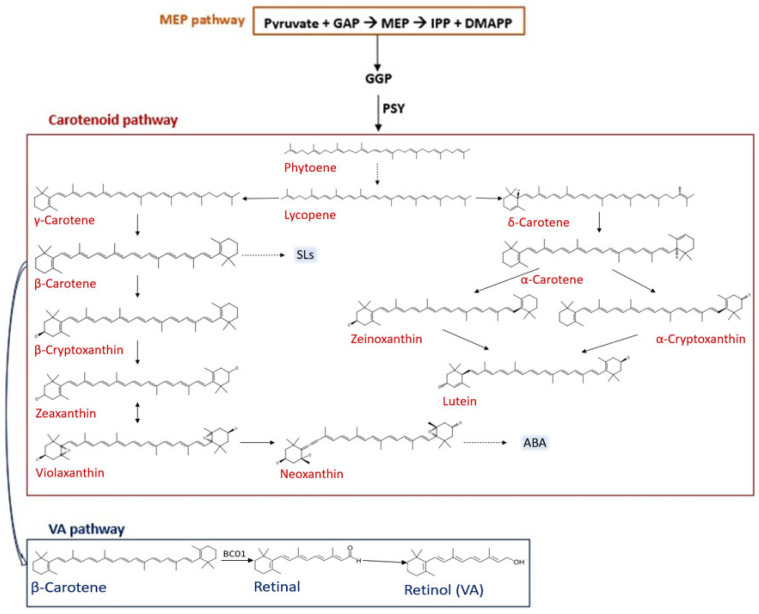
Simplified carotenoid biosynthetic pathway in tomato fruits (GAP, Glyceraldehyde 3-phosphate; MEP, 2-C-methyl-D-erythriol 4-phosphate; IPP, Isopentenyl diphosphate; DMAPP, dimethylallyl diphosphate; GGP, geranylgeranyl diphosphate; PSY, phytoene synthase; SLs, strigolactones; ABA, abscisic acid; VA, vitamin A).

The metabolic route originates from isoprenoid precursors and involves a series of enzymatic reactions leading to the formation of major carotenoid compounds, including phytoene, phytofluene, β-carotene, and lycopene. Figure 2 illustrates, in a simplified form, the key steps and enzymes responsible for the accumulation of these pigments during fruit ripening.

However, a diet rich in fruits and vegetables containing carotenoids does not guarantee their nutritional efficiency. The structure of the food matrix, processing methods and fat intake influence their bioavailability. Due to their lipophilic nature, the absorption of carotenoids is increased when they are consumed together with dietary lipids. Also, mechanical fragmentation processes, such as chopping or homogenization, along with thermal treatments can destabilize the plant cell structure, favouring the release and solubilization of carotenoids. Thus, processed tomato products, such as sauces, pastas or juices, may have a higher bioavailability compared to fresh tomatoes [5,23].

The carotenoid composition and overall nutritional quality of tomatoes are influenced by several factors, including variety, ripening stage, environmental conditions, and cultivation practices, which can significantly affect the content of nutrients and bioactive compounds [12]. The diverse carotenoid composition of tomatoes, combined with their high global consumption, makes this fruit an important dietary source of bioactive compounds associated with potential health benefits.

Carotenoids are unevenly distributed within tomato tissues. Thus, the seeds and skin have a higher concentration of these pigments, which makes them valuable by-products for carotenoid extraction [24].

β-carotene represents a smaller part of the total carotenoids in tomatoes, having a symmetrical structure characterized by the presence of two b-ionic rings, which allows its biotransformation into retinol (vitamin A) in the human small intestine. Its nutritional importance and health effects have been further highlighted in recent meta-analyses on the efficacy of carotenoids. β-carotene also supports essential physiological functions, regulating the immune response, maintaining epithelial integrity, contributing to the prevention of skin oxidation and the harmful effects of ultraviolet radiation, while acting as a powerful antioxidant [5,25].

Tomatoes generally contain relatively low β-carotene content (1–5% of total carotenoids) [8], compared with richer sources such as carrots, sweet potatoes, and pumpkin, which are well-recognized dietary sources of this provitamin A carotenoid [5]. Even though tomatoes provide only about 0.06–1.2 mg/100 g FW [26], their high global consumption frequency makes them a nutritionally relevant source of β-carotene in many diets [27], with processing enhancing its extractability and bioaccessibility. For instance, Molteni et al. [28] presented that β-Carotene derived from papaya exhibited nearly three times greater bioavailability than that obtained from carrots and tomatoes, while the two matrices (carrots and tomatoes) showed a negligible difference. Processing induces the formation of cis isomers such as (13Z)-β-carotene, which improves the micellar incorporation [8]. Moreover, novel green technologies like ultrasound, high-pressure homogenization, and supercritical CO_2_ have been shown to significantly increase the extraction yields of β-carotene from tomato matrices. Advanced recovery techniques (e.g., pulsed electric fields, high-pressure homogenization) applied to tomato industrial residues also efficiently release β-carotene [29].

The molecule is recognized for also for its antioxidant and anti-inflammatory roles, which include the modulation of gene expression [30,31], and the interaction with multiple molecular targets and signalling pathways (e.g., NF κB, JK2/STAT3) to mitigate inflammatory damage and oxidative stress in cell and animal models [32]. Regarding its immune function, it can modulate immune cell proliferation, differentiation, and cytokine production and fine-tuning immune homeostasis [31].

Minor carotenoids such as phytoene and phytofluene, colourless precursors in the carotenoid biosynthetic pathway, are consistently present and contribute to the overall antioxidant potential, despite their lower concentrations [33]. Despite their lack of visible pigmentation, both compounds are efficiently absorbed and accumulate in human plasma. Reported median plasma concentrations in healthy adults are approximately 0.16 μmol/L for phytoene and 0.05 μmol/L for phytofluene, reflecting regular dietary intake of carotenoid-rich fruits and vegetables such as tomatoes and carrots [34]. Beyond their structural role in carotenoid biosynthesis, phytoene and phytofluene possess biological activity in humans based on their conjugated double bonds that enable the absorption of UV-A and UV-B radiation, contributing to photoprotective properties, particularly in skin tissues. Experimental studies suggest additional antioxidant and anti-inflammatory effects, making them promising agents in the field of dietary prevention of oxidative stress–related conditions [35].

Furthermore, dietary assessments in various regions have highlighted processed tomato products as major sources not only for coloured carotenoids like lycopene but also for phytoene and phytofluene, underlining their nutritional relevance [33].

Neurosporene is an intermediate in the biosynthesis of lycopene from ζ-carotene and is commonly detected in moderate concentrations in some tomato varieties. Recent studies have shown an association between the presence of neurosporene in the pulp of red cherry tomatoes and significant antiproliferative activity on some tumor cell lines. These observations suggest that the accumulation of neurosporene may indicate an intense metabolic activity of carotenoids during the ripening process and could contribute to the bioactive potential of tomatoes [36].

In tomatoes, there are also present low concentrations of lutein and zeaxanthin, but recent advances show that their levels could be significantly increased. Metabolic engineering has enabled the development of tomato lines with enhanced lutein and zeaxanthin accumulation, positioning them as valuable dietary sources for eye health [37]. While lycopene dominates in both quantity and antioxidant potential, other compounds, such as β-carotene, phytoene, lutein, and zeaxanthin, play complementary roles in supporting human health.

Nonetheless, prioritizing lycopene from tomatoes as the main compound is justified due to its key contribution to antioxidant activity and its significant role in oxidative stress modulation, having superior bioavailability and a well-documented correlation with protective effects on cardiovascular health and the prevention of certain types of cancer, unlike other secondary compounds with limited impact or insufficiently supported by clinical data. Moreover, most dietary supplements based on carotenoids derived from tomatoes are primarily formulated with lycopene, which confirms its biological relevance and practical applicability.

### 2.2. Lycopene

#### 2.2.1. Chemical Structure

When it comes to fully ripened red tomatoes, lycopene dominates the carotenoid profile and has been widely investigated for its antioxidant and potential anti-inflammatory effects [38].

Lycopene is an acyclic hydrocarbon carotenoid (C_40_H_56_) whose structure contains an extensive system of conjugated double bonds, responsible for both its deep red colour and its well-documented antioxidant properties. This structural configuration allows it to efficiently deactivate singlet oxygen and scavenge reactive oxygen species, thus contributing to protection against oxidative stress [39].

Through these mechanisms, lycopene contributes to the protection of essential biomolecules, including lipids, proteins, and nucleic acids, from oxidative stress, a process strongly implicated in the pathogenesis of chronic diseases such as atherosclerosis and cardiovascular dysfunction. Among dietary carotenoids, lycopene has emerged as one of the most potent dietary antioxidants. Several experimental studies have reported higher antioxidant activity compared with certain other carotenoids and lipid-soluble antioxidants under specific experimental conditions [40].

In lipid peroxidation assays and cellular models, lycopene has been shown to significantly reduce levels of malondialdehyde (MDA), a marker of lipid peroxidation, and to enhance the activity of endogenous antioxidant enzymes, such as superoxide dismutase (SOD), glutathione peroxidase (GPx), and catalase (CAT) [9]. For example, Wang S. et al. [41] reported that dietary lycopene supplementation in broiler chickens increased hepatic and serum levels of SOD and GPx, as well as upregulated the expression of genes in the Keap1-Nrf2 antioxidant pathway. Similarly, Albrahim [42] reported in a rat model that lycopene supplementation significantly enhanced the hepatic antioxidant enzyme activities, including SOD, CAT, and GPx, while lowering lipid peroxidation markers such as MDA. These findings suggest that lycopene may contribute to the modulation of endogenous antioxidant defences and oxidative stress responses.

While numerous in vitro and animal studies have reported beneficial antioxidant effects of lycopene, these findings cannot be directly extrapolated to humans. Differences in absorption, metabolism, tissue distribution and pharmacokinetic behaviour may affect the biological response observed in human subjects. In addition, experimental studies often use concentrations or doses that are difficult to achieve through normal dietary intake. These factors may contribute to the inconsistencies sometimes observed between preclinical and clinical studies. Therefore, further human intervention studies are required to better understand the extent to which the mechanisms identified in experimental models are relevant under physiological conditions [5,43].

In addition to the biological activity of lycopene itself, oxidation and enzymatic cleavage processes may generate a variety of metabolites known as apo-lycopenoids. Several studies have suggested that these compounds may exert biological effects distinct from those of the parent molecule, including potential roles in antioxidant defence, cellular signalling, and gene regulation. However, the extent to which the reported health benefits of lycopene are mediated by the parent compound or by its metabolites remains unclear. A better understanding of the formation, bioavailability, and biological activity of apo-lycopenoids is needed to clarify their contribution to the overall effects attributed to lycopene [44].

#### 2.2.2. Natural Sources, Processing and Bioavailability

Tomatoes are the primary global source of dietary lycopene, accounting for more than 80% of total intake in most populations [12]. Lycopene concentrations in fresh tomatoes often fall between 2.5 and 8 mg per 100 g fresh weight, depending on variety, maturity, and growing conditions; some cultivars under optimal ripeness have shown values around 4 to 5 mg/100 g FW [42,43].

Table 1 presents the content of lycopene in other food sources.

Although fruits like gac, watermelon, papaya, grapefruit, and guava can contain comparable or even higher lycopene levels per 100 g, tomatoes, especially processed tomato products, remain the most practical source.

Meanwhile, dietary surveys estimate that over 80% of total lycopene intake in populations such as Korea comes from tomatoes and their processed derivatives, including ketchup and juice [48]. These factors, including high consumption, year-round availability, and the improved bioavailability associated with processed tomato products, contribute to the central role of tomatoes in dietary lycopene intake worldwide.

Thermal processing methods, including heating and boiling, can increase the bioaccessibility of lycopene by promoting its release from the plant cellular matrix and inducing the conversion of all-trans lycopene into cis-isomers. In fresh tomatoes, lycopene occurs predominantly in the all-trans configuration, whereas thermal treatment and metabolic transformations may increase the proportion of cis-isomers. Because cis-isomers exhibit greater solubility in mixed micelles than the all-trans form, they are generally considered more readily absorbed during digestion [42]. Moreover, a randomized crossover clinical trial demonstrated that lycopene from cis-rich tangerine tomato juice was absorbed approximately 8.5 times more efficiently than lycopene from conventional red tomato juice, further supporting the enhanced bioavailability of cis-isomers [52].

Following release from the food matrix, lycopene becomes incorporated into mixed micelles formed in the presence of bile salts and dietary lipids. This micellar incorporation is a critical step for intestinal uptake, as lycopene is a highly lipophilic compound. Once absorbed by enterocytes, lycopene is incorporated into chylomicrons and transported through the lymphatic system before entering the bloodstream. From there, it is distributed to various tissues, with particularly high concentrations reported in the liver, adrenal glands, testes, and prostate. The efficiency of this process may be influenced by several factors, including food matrix composition, dietary fat intake, individual metabolic differences, and the relative proportion of cis- and trans-isomers present in the consumed product [53]. Studies in both animal and human models have shown higher concentrations of cis-lycopene in plasma and tissues than would be expected from its proportion in the diet, suggesting the involvement of post-digestive isomerization and selective absorption mechanisms. Recent evidence continues to support the view that cis-isomers exhibit greater bioavailability than the all-trans form due to their enhanced solubility in mixed micelles and more efficient incorporation into chylomicrons [43,54].

The presence of dietary lipids further enhances lycopene bioavailability. Heat and presence of lipids (e.g., cooking with oil) facilitate the conversion of all-trans-lycopene into cis-forms, for example, a study of Pathak and Sagar (2023) [55] demonstrated that by heating the tomato pulp with about 10% added oil, at 120 °C, increased the proportion of cis-lycopene by nearly 40% compared to the unheated controls. Yan et al. [56] conducted an in vitro digestion study and assessed that unsaturated fatty acids significantly increased the micellization and cellular uptake of lycopene during simulated small-intestinal digestion.

Lycopene, being lipophilic, requires dietary fats and bile for optimal absorption. Other study has highlighted that at least 5–10 g of fat is necessary in a meal to enhance the lycopene uptake through micelle formation [43].

Clinical evidence showed that a regular intake of tomato paste or sauce can lead to meaningful reductions in low density lipoprotein (LDL) cholesterol, systolic blood pressure, and oxidative stress markers, driven by the enhanced uptake of bioactive lycopene [57,58].

Nevertheless, the magnitude of the bioaccessibility advantage associated with processed tomato products may vary considerably among studies. Factors such as processing conditions, cultivar, food matrix composition, lipid content, and interindividual variability may all influence lycopene absorption and metabolism, making direct comparisons between studies challenging [59].

Although numerous studies have reported enhanced lycopene bioavailability from processed tomato products, the available evidence is not entirely consistent. While some studies report higher lycopene bioavailability in processed tomato products compared to fresh tomatoes, others have shown that excessive thermal treatment may lead to carotenoid degradation, highlighting inconsistencies across experimental conditions. Differences in processing methods, food matrix composition, lipid content, cultivar, and study design may contribute to variability in reported outcomes. While thermal processing generally promotes lycopene release from the plant matrix and increases the proportion of bioavailable cis-isomers, excessive processing conditions may also lead to degradation of carotenoids and loss of other bioactive compounds. Consequently, the relationship between processing and lycopene bioavailability appears to be influenced by multiple factors, and direct comparisons among studies should be interpreted with caution [54,60].

**Figure 3:**
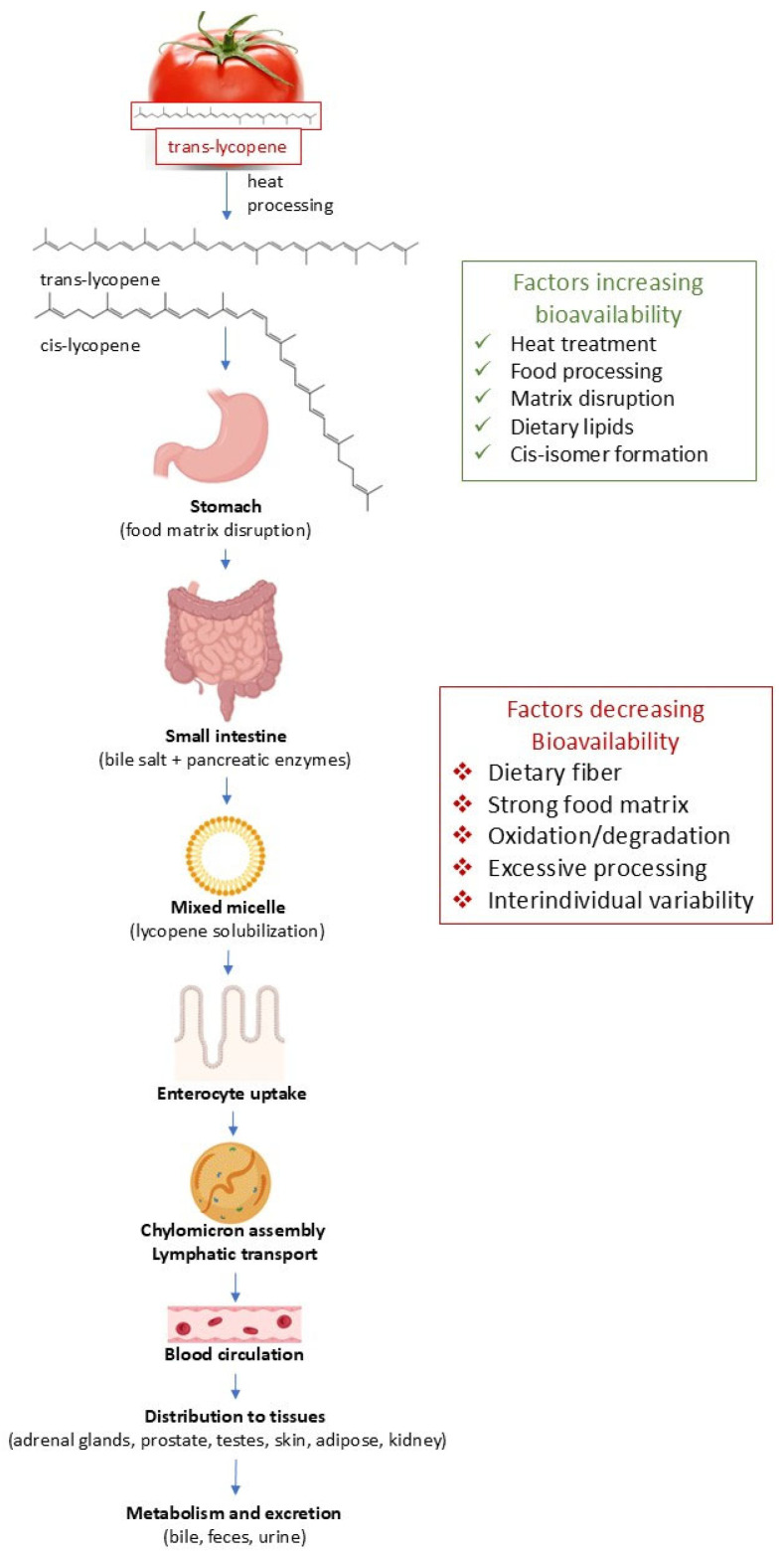
Schematic representation of lycopene isomerization, digestion, absorption and factors influencing bioavailability. Created by the authors based on information reported in [43,61].

A schematic overview of the processes affecting lycopene bioavailability is presented in Figure 3.

## 3. Extraction and Analysis Methods

Efficient extraction and precise quantification of lycopene from tomatoes are critical for determining their potential in food, nutraceutical, and pharmaceutical applications. Traditional organic solvent-based techniques, although widely used, often involve environmental and safety concerns due to high solvent consumption and prolonged processing times. To address these challenges, modern extraction technologies—such as ultrasound-assisted, microwave-assisted, enzyme-assisted, and supercritical fluid extraction have emerged as more sustainable, selective, and energy-efficient alternatives, ensuring better preservation of bioactive compounds.

Given the fact that lycopene is a lipophilic pigment localized mostly in tomato skins and pulp, its extraction benefits from non-polar or moderately polar solvent systems. Contemporary techniques outperform conventional methods on yield, speed, and environmental footprint.

Microwave-assisted extraction (MAE) is an unconventional method that uses microwave energy to accelerate mass transfer and increase extraction yield in a reduced time. Chada et al. [62] investigated the valorization of tomato pomace by applying unconventional extraction techniques, namely microwave-assisted extraction (MAE) and pressurized liquid extraction (PLE), after an innovative spouted bed fluidized bed drying pretreatment, optimizing the process parameters (temperature, ethanol:ethyl acetate solvent composition, extraction time for MAE and flow rate for PLE), showing that MAE at 90 °C for 3 min ensured the highest lycopene recovery, while PLE led to extracts with superior antioxidant activity, highlighting the efficiency of unconventional techniques. Wu et al. [63] optimized the extraction of lycopene from tomato powder by a microwave-assisted method using ionic liquids (IL–MAE), evaluating the influence of microwave power, extraction time, ionic liquid concentration, and liquid/solid ratio.

Ultrasound Assisted Extraction (UAE) leverages acoustic cavitation to disrupt the cell structures and enhance solvent penetration. Li et al. [64] investigated the use of ultrasound-assisted extraction as an intensive method for the recovery of lycopene from plant matrix, demonstrating the applicability of this technique in obtaining extracts rich in bioactive compounds, subsequently characterized and quantified by high-performance liquid chromatography (HPLC). High intensity ultrasound combined with natural oils (e.g., extra virgin olive, grape, peanut) allowed the lycopene extraction from organic waste, achieving satisfactory yields while reducing conventional solvents. Olive oil under agitation performed better, though caution is advised due to the potential oxidation at high ultrasound intensity [65]. Another study combined ultrasounds with volatile natural deep eutectic solvent (VNADES) menthol: thymol (1:1 for the recovery of lycopene from tomato peel waste, demonstrating the applicability of this sustainable approach as an alternative to conventional solvents, and the extracts obtained were subsequently characterized and quantified by high-performance liquid chromatography [66]. Similarly, Mozafari et al. [67] investigated the extraction of lycopene from tomato by-products using pulsed ultrasound-assisted extraction with ethanol as the green solvent, applying Response Surface Methodology (RSM) to evaluate and model the influence of temperature, time, and amplitude on the extraction yield of lycopene and β-carotene, establishing the optimal process conditions to maximize the recovery of bioactive compounds. In another study, Drosou et al. [68] evaluated the valorization of tomato by-products by applying the Soxhlet, UAE–MAE and PLE methods, optimizing the solid/liquid parameters, microwave power and ultrasound power in the case of peels through Response Surface Methodology (RSM). Among the remarkable achievements are the obtaining of a high lycopene content from the peels (37.08 mg/100 g) with an extraction yield of 91.20% by the synergistic UAE–MAE method in just 8 min, as well as the efficient recovery of oil from the seeds. Similarly, Badea et al. [15] used USE with natural eutectic solvents based on choline chloride and 1,3-butanediol (1:5), systematically evaluating the influence of the solid/liquid ratio, extraction time, temperature and ultrasonic power level on the yield and antioxidant capacity, establishing the optimal conditions at 65 °C, 12 min and 70% ultrasonic power, which led to the obtaining of an extract rich in lycopene, β-carotene and tocopherol, with high antioxidant activity.

Supercritical Fluid Extraction (SFE) using supercritical CO_2_ (often with ethanol as a co-solvent) provides selective extraction under mild thermal conditions and avoids residual solvents. Mihalcea et al. [69] extracted lycopene-rich oleoresins from tomato peels via SC-CO_2_, achieving fractions where lycopene accounted for 93% and 76% of the total carotenoids in different separators.

Recent studies highlight also the use of enzyme-assisted extraction (EAE) as an eco-friendly approach to recover lycopene from tomato by products. In particular, the use of cellulase, pectinase, and hemicellulase enzymes facilitates the hydrolytic breakdown of cell wall polysaccharides, thereby increasing the cell wall permeability and promoting enhanced lycopene release. This enzymatic pretreatment results in higher extraction yields, shorter processing times, and reduced reliance on organic solvents. Furthermore, combining cellulolytic and pectinolytic enzymes (e.g., Celluclast with Pectinex) under optimized conditions (e.g., 40 °C, 5 h, enzyme: substrate ratio 0.2 mL/g) produced the highest yields when followed by solvent extraction [70]. The study of Pathak and Sagar [55] reported that using pectinase on tomato waste led to up to a 10-fold increase in lycopene content compared to untreated samples, underscoring the remarkable effect of enzymatic hydrolysis. Scaglia et al. [71] improved the extraction of lycopene from tomato by an enzymatic pretreatment with pectinase and cellulase, followed by a rapid extraction procedure (FEP) with ethyl acetate, a method that allowed to increase the recovery of lycopene and simultaneously obtaining valuable oil and carbohydrate fractions. Another study followed the extraction of lycopene that was preceded by an enzymatic treatment using commercial preparations based on pectinases, cellulases and hemicellulases (Lallzyme EX-V), which facilitated the degradation of the cell wall of tomato peels and led to a significant increase in yield in the subsequent ultrasound-assisted extraction step [72].

The integration of modern extraction technologies—MAE, UAE, SFE and EAE—demonstrates significant potential in maximizing the yield and quality of lycopene recovered from tomatoes and their by-products, offering sustainable, efficient and scalable alternatives to conventional methods, with extensive applicability in the food, nutraceutical and pharmaceutical industries. Over the past decade, analytical strategies for lycopene determination in dietary supplements such as tablets, capsules, and syrups have evolved to meet regulatory quality control requirements. Reversed-phase HPLC coupled with UV or diode-array detection (DAD) continues to be the reference method for routine label-claim verification, with studies reporting high linearity (r^2^ > 0.995), accuracy, and precision when validated according to ICH guidelines [73]. Method development has focused on adjusting mobile phase composition, flow rates, and detection wavelengths (≈470–475 nm) to ensure selective and reliable quantification within complex multicarotenoid formulations. Singh et al. [74] developed and validated an RP-HPLC method for the simultaneous determination of lutein, lycopene and β-carotene from tablets, demonstrating excellent linearity (R^2^ = 0.998 for lycopene), high precision (%RSD < 2%) and recoveries close to 100%.

To provide a comprehensive overview of the approaches reported in the literature, the main lycopene extraction methods from tomatoes and tomato by-products, together with the corresponding extraction parameters, operational conditions, and analytical techniques used for extract characterization, are summarized in Table 2.

## 4. Compound Stability Considerations

Lycopene exhibits pronounced instability when exposed to heat, light, and oxygen, undergoing degradation and isomerization under such stressors. To minimize these losses during extraction and handling, recent protocols recommend shielding samples from light and oxygen and performing operations at moderate temperatures (e.g., ≤40–50 °C) and under inert atmospheres [49].

Numerous microencapsulation and nanoencapsulation systems have been developed for the stabilization and delivery of lycopene, aiming to improve the stability, solubility, and bioavailability of this lipophilic carotenoid. Polymeric nanoparticles based on polylactic-co-glycolic acid (PLGA) have demonstrated the ability to protect lycopene against oxidative degradation and improve antioxidant stability, being used especially for dermato-cosmetic applications and controlled delivery systems [78,79]. Also, biopolymer-based systems, such as nanoparticles formed by electrostatic complexation between polysaccharides and proteins or chitosan-alginate nanocapsules, can increase the dispersibility in aqueous media and the antioxidant stability of lycopene, contributing to increasing its bioavailability in nutraceutical and pharmaceutical applications [80,81]. Vesicular systems such as niosomes, obtained from nonionic surfactants and cholesterol, offer good thermal stability and the possibility of controlled release of the active compound, and are frequently investigated for topical applications [82]. Formulations based on nanoemulsions or self-emulsifying systems have been shown to be effective in improving the solubility and bioaccessibility of lycopene in the gastrointestinal tract, due to the fine dispersion of the lipid phase in the aqueous medium [83]. In addition, the transformation of nanoemulsions into powders by techniques such as spray-drying, using coating agents such as maltodextrin, allows the obtaining of stable ingredients, easy to integrate into food or nutraceutical products [84]. Nanoemulsions can also be used in cosmetic formulations, where the small particle size favors cutaneous penetration and increases the photoprotective efficiency of lycopene [85,86].

Depending on the coating materials and the technique used, the particle size reported in these systems ranges from approximately 100 nm to several hundred nanometers, and the encapsulation efficiency can frequently exceed 80–90%. These results highlight the high potential of encapsulation systems for protecting lycopene and for expanding its applicability in the food, pharmaceutical and cosmetic fields.

An overview of the main encapsulation methods used for lycopene stabilization and delivery is presented in Table 3.

Comparing the studies, it is highlighted that the type of encapsulation system and the materials used significantly influence the particle size, stability and encapsulation efficiency of lycopene. In general, the analyzed systems present dimensions in the nanometric range and high encapsulation efficiencies, which contribute to protecting the compound and improving its performance in the targeted applications. These results suggest that encapsulation technologies can improve the stability of lycopene and expand its potential applications in food, pharmaceutical, and cosmetic products.

Beyond encapsulation efficiency and immediate stability improvements, long-term storage performance remains an important consideration for the practical application of lycopene delivery systems. Several studies have shown that temperature, light exposure, oxygen availability, and humidity can significantly affect lycopene stability during storage. Although nanoencapsulation generally improves the protection of lycopene against oxidation and degradation, progressive losses may still occur under prolonged storage conditions. Recent studies have highlighted that storage conditions play a critical role in preserving the antioxidant activity and chemical integrity of encapsulated bioactive compounds, emphasizing the importance of shelf-life evaluations under realistic environmental conditions. Therefore, long-term stability studies remain essential for assessing the industrial applicability of lycopene-based formulations [87,88].

Despite these advantages, the industrial implementation of nanoencapsulation technologies remains challenging. Many nanoencapsulation systems have demonstrated excellent performance at laboratory scale; however, large-scale production requires careful consideration of process complexity, equipment costs, energy consumption, regulatory compliance, and batch-to-batch reproducibility. In addition, the economic feasibility of certain nanoencapsulation approaches may limit their commercial application, particularly when expensive carrier materials or specialized processing technologies are required. Consequently, future research should focus not only on improving encapsulation efficiency and bioavailability but also on developing cost-effective and scalable production strategies suitable for industrial implementation [89,90].

## 5. Dietary Intake Versus Supplemental Intake

Evidence increasingly points to a “food first” message. Tomato matrices deliver lycopene alongside other phytochemicals (e.g., β-carotene, phytofluene, phenolics), which may act additively or synergistically while heat processing and lipid carriers also enhance the absorption. By contrast, capsule supplements often provide all-trans lycopene without the same matrix or isomer distribution, which might partly explain why short-term trials on PSA are neutral despite observational signals from diets rich in tomato products [91].

### 5.1. Safety and Practical Implications

In terms of safety, lycopene from food sources is considered safe, and the doses used in clinical trials, typically between 10 and 30 mg/day, are generally well tolerated. However, until more robust causal evidence is available, routine supplementation with high doses of lycopene for cancer prevention cannot be recommended. Future research should focus on longer-term randomized clinical trials that use validated biomarkers of exposure (e.g., cis-isomers of lycopene) as well as intermediate indicators of cancer biology, such as IGF-1 signaling or inflammatory mediators in target tissues. It is also important to evaluate formulations that replicate the bioavailable food matrix of tomato products [91,92].

Lycopene supplements in human studies often range from 2 to 20 mg/day, and in some context doses of 30 mg/day or higher have been explored without serious adverse effects. In one adjunctive trial involving *H. pylori* therapy, a 30 mg/day lycopene dose was tested [93]. Bioavailability is significantly higher when lycopene is consumed in processed tomato products and alongside dietary fats. Heat processing induces cis-isomerization, which further enhances absorption and tissue distribution [43]. These pharmacokinetic characteristics are particularly relevant when considering the administration or delivery of lycopene to the stomach. Studies in animal models show that very high doses of lycopene, in the context of acute exposure to high amounts of ethanol, can paradoxically aggravate gastric mucosal damage [94]. Although the direct extrapolation of these results to humans remains uncertain, these observations suggest the need for a cautious approach, avoiding excessive consumption of lycopene supplements in situations of acute alcohol-induced stress, until these effects are clarified by further clinical studies.

Overall, the current evidence available in the open-access literature outlines a coherent theory that lycopene has the ability to neutralize free radicals, reduce inflammatory signaling, stimulate Nrf2-dependent cytoprotective mechanisms, and modulate gastric secretions. These effects may plausibly contribute to the protection of the gastric mucosa. However, human data remain limited and inconclusive. Recent clinical trials have not consistently demonstrated either ulcer healing or eradication of *Helicobacter pylori* infection, and population-based studies do not reveal a clear signal of protection against gastric cancer. For now, lycopene-rich foods—particularly processed tomato products—could be encouraged as part of a balanced diet, but supplements aimed specifically at ulcer or gastritis therapy remain investigational.

### 5.2. Lycopene Formulations

Standardization of formulations containing bioactive compounds is essential to ensure the quality, safety and reproducibility of biological effects, as well as to facilitate the comparability of results between different studies and commercial products [19].

Standardized lycopene formulations refer to preparations with a defined and analytically verified lycopene content, typically expressed as milligrams per serving (e.g., 10–30 mg/capsule). Standardization ensures batch-to-batch consistency, chemical stability, and predictable bioavailability. Because lycopene is a highly unsaturated, oxidation-sensitive carotenoid, these formulations are engineered with stabilizing agents and delivery matrices that protect against degradation by oxygen, light, and heat [95].

In the nutraceutical field, the development of standardized formulations has become an important approach for ensuring the quality and consistency of bioactive compounds. This trend is particularly obvious in the United States, where the dietary supplement industry widely uses analytically characterized lycopene preparations with defined dosage ranges. In these products, lycopene is commonly incorporated into softgel formulations that improve the stability and intestinal absorption of this highly lipophilic carotenoid [25,42]. As a result, research conducted in the United States has contributed significantly to establishing reference dosage ranges, obtaining bioavailability data, and developing clinical evaluation frameworks for lycopene supplementation [9].

In the United States, lycopene has long been studied as a nutraceutical ingredient, being used especially in dietary supplements intended to support cardiovascular and metabolic health and in the context of the prevention of certain oncological diseases. American research has mainly focused on purified or synthetic lycopene preparations, formulated as capsules, softgels or oil-based systems, designed to improve the bioavailability of this highly lipophilic carotenoid. Such supplements are frequently investigated in clinical and translational studies that analyze parameters such as oxidative stress, inflammatory processes and lipid metabolism, highlighting the significant antioxidant role attributed to lycopene [47,92].

Recent research in Europe is increasingly focused on the development of multifunctional antioxidant complexes, in which standardized lycopene is combined with polyphenols from olives or grapes, with the aim of exploiting possible synergistic effects on oxidative stress and inflammatory processes [60]. At the same time, future directions in formulation are aiming at the integration of more sustainable technologies, such as ecological extraction methods, biopolymer encapsulation systems and controlled release structures, aimed at improving both the stability of bioactive compounds and their efficacy.

Currently, standardized formulations containing only lycopene, are considered an important benchmark for clinical consistency and compliance with regulatory requirements in the European Union. These products provide a precise amount of lycopene, typically expressed in milligrams per dose, and have well-documented safety and bioavailability profiles, being frequently used in interventional studies investigating cardiovascular, oxidative, and inflammatory biomarkers [96,97].

On the other hand, complexes combining lycopene with polyphenols have begun to attract increasing interest due to their enhanced antioxidant and anti-inflammatory potential. The association of lycopene with olive-derived polyphenols, for example fractions rich in hydroxytyrosol, may contribute to a more efficient intestinal handling of the carotenoid, facilitating emulsification processes and protecting lycopene molecules against oxidative degradation during digestion. Recent studies conducted in a European context on tomato-based supplements enriched with olive polyphenols have shown improvements in some indicators associated with oxidative stress, and research conducted on human subjects who used olive oil enriched with lycopene reported favorable effects on lipid oxidation and inflammatory markers. However, clinical confirmation of these effects remains dependent on the specific type of formulation and requires further studies on a larger scale [60,98,99].

Overall, although standardized formulations containing only lycopene offer the advantage of reproducible dosing and a well-characterized safety profile, lycopene–polyphenol hybrid systems represent an emerging category of functional products with the potential to improve both compound stability and biological efficacy. In this context, future studies conducted in the European space should integrate detailed compositional analyses, in vitro digestion models and clinical bioavailability assessments to validate the proposed synergistic benefits.

At the same time with these developments at European level, the Romanian nutraceutical sector has experienced a significant development of supplements containing lycopene and polyphenols, reflecting global trends oriented towards the use of natural bioactive compounds to support cardiovascular, metabolic, dermatological and immune system health. Lycopene, a lipophilic carotenoid with remarkable antioxidant and anti-inflammatory activity, is frequently associated in these products with polyphenols such as quercetin, resveratrol, catechins or phenolic acids, in order to enhance therapeutic effects through additive or synergistic modulation of oxidative stress, inflammation and endothelial function [9,100].

To illustrate the diversity of lycopene-based nutraceutical formulations across different markets, representative commercial supplements available in the United States, Europe, and Romania are summarized in Table 4.

### 5.3. Regulatory Safety Benchmarks

In the United States, dietary supplements are regulated by the Food and Drug Administration (FDA) under the Dietary Supplement Health and Education Act (DSHEA) of 1994, which defines supplements as products intended to supplement the diet and containing vitamins, minerals, herbs, or other bioactive compounds. Unlike drugs, supplements do not require prior FDA approval for marketing, and the responsibility for safety, quality, and proper labeling rests with the manufacturer. However, production must comply with Current Good Manufacturing Practices (cGMP; 21 CFR Part 111), and the introduction of a New Dietary Ingredient (NDI) must be notified to the FDA. There is no official recommended daily allowance (RDA) for lycopene in the United States; however, clinical trials and commercial products frequently use doses of approximately 5–30 mg/day, which are considered safe in the context of dietary supplementation [9,116].

At European level, food supplements are regulated by a harmonised legislative framework that includes Directive 2002/46/EC [117] on food supplements, as well as Regulation (EC) No 1924/2006 [118], which establishes that nutrition and health claims must be supported by sound scientific evidence and assessed at European Union level. Regarding the safety of lycopene, the European Food Safety Authority (EFSA) has established an acceptable daily intake (ADI) of 0.5 mg/kg body weight/day for lycopene used as a food additive (E160d), a value maintained in subsequent safety assessments of this compound. For an adult with a body mass of approximately 70 kg, this corresponds to an estimated intake of approximately 35 mg/day, taking into account both the contribution from food additives and natural dietary sources [119]. Given the highly lipophilic nature of lycopene, modern formulations frequently use lipid-based delivery systems, and recent research is exploring nanoformulations and nanoencapsulation systems to improve the bioavailability and stability of bioactive compounds [120].

In Romania, the regulation of food supplements is aligned with European legislation through the transposition of Directive 2002/46/EC [108] and through normative acts such as the Order of the Ministry of Health no. 1069/2007 [121]. Supplements must be notified to the competent authorities before being marketed, and their composition and labeling must comply with European requirements regarding ingredients and health claims. Although there is no official recommended daily dose for lycopene, supplements available on the European and Romanian market usually contain 10–30 mg of lycopene per daily dose, values considered compatible with the safety limits established by EFSA.

Despite the growing availability of lycopene-containing dietary supplements, several aspects should be considered when evaluating their quality, safety, and potential effectiveness. Regulatory requirements for food supplements differ from those applied to medicinal products, and manufacturers are generally not required to demonstrate clinical efficacy before marketing. Consequently, the composition and performance of commercially available products may vary depending on the source of lycopene, extraction procedures, formulation strategies, and storage conditions [122].

Product standardization is particularly important, as differences in isomer composition, stability, and bioavailability may influence the biological effects achieved following supplementation. In addition, insufficient quality control may increase the risk of lycopene degradation during processing and storage, potentially affecting product efficacy [123].

Particular attention should also be given to the quality and traceability of raw materials, as non-standardized products may present variability in composition and may be more susceptible to contamination with undesirable substances introduced during cultivation, processing, or manufacturing. Independent quality verification and certification procedures may help improve product reliability and consumer confidence [124].

Although lycopene is generally regarded as safe at commonly used doses, rigorous quality assurance, standardized production procedures, and appropriate regulatory oversight remain essential to ensure product consistency, safety, and consumer confidence [123].

## 6. Pharmacological Properties and Biological Activities

Lycopene, a potent antioxidant carotenoid found primarily in tomatoes and tomato products, is recognized for its extensive antioxidant and anti-inflammatory properties [5]. According to Shafe et al. [47], lycopene can reduce oxidative stress in multiple organs, including the heart, liver, kidneys, lungs, bones, eyes, and nervous system, thus contributing to the prevention of metabolic dysfunctions such as obesity and diabetes. In the context of cardiometabolic health, a review study indicates that the antioxidant and anti-inflammatory activity of lycopene makes it a promising compound for the prevention and management of metabolic syndrome, obesity, and type 2 diabetes, and is also associated with improved lipid profiles and metabolic functions [39].

Numerous experimental and epidemiological studies have investigated the potential anticancer effects of lycopene. This carotenoid may help protect DNA from oxidative damage, promote apoptosis (programmed cell death), and inhibit several biological pathways involved in tumor development. Epidemiological and experimental evidence also suggests that a higher intake of lycopene is associated with a lower risk of certain cancers, particularly prostate cancer, as well as cancers of the breast, lung, gastrointestinal tract, and liver [92].

Evidence from both experimental models and clinical investigations indicates that lycopene may contribute to improved cardiovascular and metabolic health, liver function, and a role in the prevention of certain types of cancer, due to its antioxidant and anti-inflammatory properties. However, although the results are promising, large-scale clinical trials in humans are needed to establish optimal doses and fully confirm its benefits, including in terms of mental health and neurodegenerative diseases.

### 6.1. Antioxidant Activity

Carotenoids from tomatoes, especially lycopene, are bioactive pigments recognized for their numerous potentially beneficial effects on health. The chemical structure of lycopene, characterized by a long chain of conjugated double bonds, gives it the ability to neutralize singlet oxygen and other reactive oxygen species, involved in oxidative stress processes. Some studies suggest that due to this structure, lycopene may have a higher antioxidant efficiency than other carotenoids, such as β-carotene [9,43].

This carotenoid is fat-soluble and it is found in high amounts in tomatoes, watermelon and other red fruits or vegetables. It is considered one of the most effective natural compounds in neutralizing free radicals. Recent research highlights that lycopene acts through several antioxidant mechanisms, contributing to reducing oxidative stress and protecting cells from damage.

This molecule exerts potent antioxidants through several biological mechanisms. First, it can directly neutralize reactive oxygen species (ROS), including singlet oxygen and peroxyl radicals, thereby reducing cellular damage caused by oxidative stress [125,126,127].

Second, lycopene contributes to strengthening the body’s endogenous antioxidant system. Experimental animal studies have shown that lycopene supplementation can increase the activity of important antioxidant enzymes, such as superoxide dismutase (SOD), catalase (CAT), and glutathione peroxidase (GSH-Px), while reducing the level of malondialdehyde (MDA), a marker of lipid peroxidation. An improvement in the GSH/GSSG balance, an indicator of the body’s antioxidant status, has also been observed [128].

In studies in rats with diet-induced hypercholesterolemia, lycopene administration was associated with increased levels of antioxidant enzymes in the heart and liver, including SOD, CAT, reduced glutathione (GSH), glutathione peroxidase (GPx), and glutathione reductase, along with decreased levels of MDA and nitric oxide (NO) in heart and kidney tissues [38,116]. In addition, lycopene can activate several cellular antioxidant defense mechanisms via the Nrf2 signalling pathway, which stimulates the expression of antioxidant response element (ARE)-dependent genes involved in protection against oxidative stress [9,128,129].

Lycopene also protects against lipid peroxidation and oxidative stress. Studies show that this carotenoid can reduce lipid oxidation in cell membranes and protect the intestinal epithelium under conditions of exposure to toxins, by activating the Keap1/Nrf2 signaling pathway, involved in the regulation of cellular antioxidant mechanisms [44,130].

In experimental neuronal models, lycopene has also demonstrated the ability to reduce neurooxidative stress induced by fatty acids, preventing cellular damage and the occurrence of cellular vacuolization [39].

### 6.2. Anti-Inflammatory Activity

Lycopene exhibits significant anti-inflammatory properties, acting by reducing the activity of factors and mediators involved in inflammation, such as NF-κB, COX-2, iNOS and various pro-inflammatory cytokines. In an experimental model of myocardial infarction in mice, lycopene administration reduced the expression of the cytokines IL-1β and TNF-α by inhibiting the NF-κB signaling pathway, which led to a decrease in cardiomyocyte apoptosis [125]. Several recent studies have reported that lycopene can modulate inflammatory responses in different tissues. For example, in an experimental model of LPS-induced jejunal inflammation in mice, administration of lycopene before exposure to the inflammatory agent inhibited the activation of the TLR4/NF-κB signaling pathway, involved in the production of proinflammatory cytokines. At the same time, an increase in the expression of the ZO-1 protein, an important protein for maintaining the integrity of the tight junctions of the intestinal epithelium, was observed, thus suggesting a possible protective effect on the intestinal barrier [128].

Similar results were observed in studies on mice exposed to atrazine, where lycopene reduced cardiac inflammation by decreasing the levels of COX-2, TNF-α, IL-6 and IL-1β and by increasing the anti-inflammatory cytokine TGF-β1. At the same time, it was found to block the TRAF6/NF-κB signaling axis and reduce the levels of nitric oxide (NO) and NOS activity, factors involved in inflammatory and oxidative processes [39].

The anti-inflammatory effects of lycopene are also associated with the inhibition of other cellular signaling pathways, such as PI3K/Akt/mTOR, in addition to suppressing NF-κB. Lycopene can also modulate TLR4/NF-κB-dependent pathways, which leads to a decrease in the expression of important inflammatory mediators, such as IL-1β, IL-6, TNF-α, COX-2 and iNOS, while also contributing to the stabilization of cell membranes and the regulation of molecular mechanisms involved in the inflammatory response [131].

Lycopene acts as a potent antioxidant, reducing reactive oxygen species that can degrade nitric oxide (NO) in the vascular endothelium. By limiting this process, lycopene helps maintain NO levels and improve endothelium-dependent vasodilation, a mechanism essential for blood vessel health [40,97]. In a clinical study, administration of a tomato extract providing approximately 15 mg of lycopene per day for six weeks to patients with moderate hypertension—already treated with angiotensin-converting enzyme (ACE) inhibitors or calcium channel blockers—resulted in significant decreases in systolic and diastolic blood pressure, suggesting a vasoprotective effect associated with NO-dependent mechanisms [61].

A meta-analysis of randomized clinical trials showed that lycopene supplementation at doses of at least 12 mg per day is associated with significant reductions in systolic blood pressure in people with prehypertension or hypertension. In contrast, effects on diastolic blood pressure were less consistent [61]. Other data indicate that lycopene may have a useful role in lowering blood pressure in people at cardiovascular risk, suggesting that it could be used as an adjunct in the management of hypertension [126].

Lycopene can also influence cholesterol metabolism through several mechanisms. It can inhibit the activity of the enzyme HMG-CoA reductase, the rate-limiting enzyme in cholesterol synthesis. Recent analyses indicate that lycopene can reduce the activity of this enzyme even at the transcriptional level, which leads to a decrease in cholesterol synthesis in experimental models of hyperlipidemia [38].

Lycopene has also been associated with increased expression of LDL receptors (LDL-R), facilitating the capture and elimination of LDL particles from the circulation. In inflammatory conditions, such as those experimentally induced by lipopolysaccharides (LPS), it has been observed that lycopene can increase LDL-R expression by activating the SREBP-2 signaling pathway, thus contributing to improving lipid balance [132].

In addition, lycopene can support hepatic lipid metabolism by activating the SIRT1 protein (Sirtuin 1). This subsequently regulates important transcription factors, such as PPARα and PGC-1α, involved in fatty acid oxidation and mitochondrial function, processes essential for maintaining healthy lipid metabolism [133].

Due to its potent antioxidant properties, lycopene can reduce lipid peroxidation, thereby limiting the formation of oxidized LDL and lipid peroxides, important factors involved in endothelial dysfunction and the development of atherosclerotic plaques. In endothelial cells, lycopene has been shown to inhibit the NF-κB signaling pathway, involved in inflammatory processes, and activate Nrf2-dependent antioxidant mechanisms, including the induction of the enzyme heme oxygenase-1 (HO-1), which has a protective role against oxidative stress. Clinical data obtained with a liposome-type lycopene formulation, designed to increase the bioavailability of the compound, have also shown a significant increase in serum lycopene levels, an approximately fivefold decrease in oxidized LDL, and a reduction in oxidative stress markers. These results highlight the importance of lycopene bioavailability for cardiovascular protective effects [39].

Furthermore, in patients with ischemic heart failure with reduced ejection fraction (HFrEF), lycopene supplementation was associated with improved endothelial function and reduced triglyceride levels. However, no significant changes in blood pressure, total cholesterol, LDL-cholesterol, or HDL-cholesterol were observed in these studies [97].

### 6.3. Anti-Cancer Activity

Lycopene exhibits credible anti-cancer mechanisms—antioxidant and anti-inflammatory actions, regulation of Nrf2/NF-κB signaling, modulation of cell cycle and apoptosis, and potential effects on the IGF axis. Human data are most suggestive (yet still mixed) for prostate outcomes, with inconsistent findings for colorectal and tentative support from carotenoid biomarker analyses in breast cancer. At present, the weight of evidence encourages consumption of tomato-rich foods prepared with healthy fats to optimize lycopene bioavailability, while large, well-designed trials continue to test whether lycopene itself versus the larger tomato food matrix drives the cancer risk reductions [91,92,134].

#### 6.3.1. Biological Pathways Involved in Anticancer Activity

Lycopene’s mechanism involves potent singlet-oxygen quenching and suppression of lipid peroxidation, thereby limiting oxidative DNA and membrane damage [133]. Beyond its direct antioxidant activity, lycopene modulates the Keap1/Nrf2 signaling pathway, promoting the expression of cytoprotective and antioxidant enzymes such as heme oxygenase-1 (HO-1) and NAD(P)H quinone dehydrogenase 1 (NQO1), thereby supporting cellular redox balance [40]. Recently, lycopene was shown to inhibit ferroptosis induced by the mycotoxin zearalenone in AML-12 hepatocytes and mouse liver, via activation of the AMPK/Nrf2 axis, improving cellular viability, reducing lipid peroxidation, and restoring liver structure and function [135].

Chronic inflammation is a well-established driver of tumor initiation and progression. Lycopene and its biologically active metabolites have been shown to attenuate NF-κB signaling, leading to reduced phosphorylation of NF-κB and downregulation of pro-inflammatory cytokines in both cellular and animal models. These anti-inflammatory effects are closely linked to redox balance, as the antioxidant activity of lycopene mitigates oxidative stress, thereby limiting NF-κB activation [127,136].

Multiple studies describe G0/G1 or S-phase arrest, modulation of cyclins/CDKs, and increased pro-apoptotic markers (e.g., Bax) alongside reduced anti-apoptotic proteins (e.g., Bcl-2). Lycopene has been reported to induce caspase activation and mitochondrial apoptosis in diverse cancer cell lines, while exerting limited cytotoxicity on non-transformed cells [137,138].

Furthermore, preclinical evidence suggests that lycopene may attenuate insulin-like growth factor (IGF) signalling a pathway relevant in colorectal and prostate carcinogenesis, and may also interfere with pro-angiogenic processes such as VEGF-mediated endothelial activation, as well as invasion-related matrix remodelling and EMT-associated changes, thereby potentially slowing tumour growth and dissemination [91,136].

#### 6.3.2. Human Evidence by Cancer Site

Tomatoes and lycopene have long been studied for prostate cancer (PCa). Observational syntheses commonly report inverse associations between higher lycopene intake or circulating levels and PCa risk or progression indicators, though heterogeneity is high [91,94]. However, randomized controlled trials focusing on surrogate endpoints generally show little to no effect of lycopene supplementation on PSA, as summarized in meta-analyses [139]. An epidemiology update likewise concluded that the evidence for a protective role remains mixed and method-dependent [139] yet inconclusive, with stronger and more consistent associations observed for dietary intake and circulating lycopene biomarkers than for outcomes from short-term supplementation trials [94,139,140].

In terms of colorectal cancer (CRC), the data is not consistent. Some animal studies support IGF-related and anti-inflammatory actions in the colon [81], but meta-analyses of human intake have found no clear association between lycopene consumption and CRC risk. Newer studies depicted inverse associations for tomato-rich diets, yet lycopene’s independent contribution remains uncertain [141]. Overall, the evidence remains equivocal, highlighting the need to distinguish foods, patterns, and biomarkers from isolated supplements [92,141].

For breast cancer, several circulating carotenoids (including lycopene) were examined and the results suggested inverse associations with risk in some analyses, but the specificity to lycopene is hard to isolate and confounding by other carotenoids and diet quality [142]. Other data provided the biological plausibility via Nrf2, NF-κB, and apoptosis pathways [131].

The overall cancer incidence and mortality in regard to carotenoids dietary intake and blood levels reported associations between higher lycopene exposure and lower overall cancer incidence and cancer mortality. However, the study designs were predominantly observational and emphasized limitations like residual confounding and exposure misclassification. As such, these findings support but do not prove an anti-cancer role for lycopene, especially as part of tomato-rich dietary patterns [134].

### 6.4. Gastroprotective Activity

One of the most well-established roles of lycopene is its ability to neutralize singlet oxygen and peroxyl radicals, thereby limiting oxidative damage to lipids and DNA. In gastric epithelial contexts challenged by Helicobacter pylori, ROS production is elevated, and antioxidant intervention reduces oxidative damage and downstream inflammatory signalling [143,144]. The emerging work on digestive precancerous lesions, lycopene is proposed to alleviate inflammation, suppress pathways such as NF-κB (including possible TLR4/TRIF involvement), and influence apoptotic responses, thus counteracting hyperproliferation induced by chronic inflammation [145,146]. Similarly, in addition to the findings on *H. pylori* and gastric precancer studies, lycopene’s biological functions indicate that it can attenuate inflammatory mediator release and downregulate eicosanoid pathways. These broader anti-inflammatory and antioxidant actions further substantiate lycopene’s plausibility as a gastric protectant [44].

Beyond its general antioxidant properties, lycopene has also been shown to activate the transcription factor Nrf2, a master regulator of endogenous defence systems. Activation of Nrf2 enhances the expression of enzymes such as superoxide dismutase (SOD), catalase (CAT), and heme oxygenase-1 (HO-1), helping to restore redox balance in mucosal and other tissues. At the same time, lycopene modulates the Nrf2/NF-κB axis, tipping the balance away from inflammatory cytokine signaling toward cytoprotection. For example, lycopene reduces IL-6 expression by upregulating NQO1 and HO-1 via Nrf2 in pancreatic acinar cells treated with ethanol/LPS [134], and in aging ovarian tissues of hens it ameliorates oxidative damage and apoptosis via activation of the Nrf2/HO-1 pathway, counteracting increased NF-κB activation with age [37].

Regarding the effects on gastric secretions and motility signaling, in a controlled mouse model of ethanol-induced gastric injury, lycopene administration reduced the volume of gastric juice and, under non-ethanol conditions, increased motilin and nitric oxide levels. While moderate doses showed improvements in oxidative and inflammatory markers, very high doses (≈150 mg/kg) exacerbated mucosal injury under ethanol stress, despite beneficial effects on some oxidative stress parameters [83]. This paradox illustrates the importance of context and dose. As such, lycopene may suppress gastric acid secretion and hinder *H. pylori* adherence to epithelial cells, but direct confirmation in humans remains scarce [47].

Despite strong data evidence, the translation of lycopene’s protective actions to human clinical outcomes remains underdeveloped. In the past years, no large randomized controlled trials (RCTs) have demonstrated that lycopene supplementation heals ulcers or resolves gastritis. The available data emphasizes that the beneficial evidence comes from small or quasi-controlled studies, which are insufficient to form definitive conclusions [47,93,145]. Thus, while laboratory evidence is persuasive, robust clinical validation is still lacking.

Abdi et al. [93] followed the complementary and dietary strategies against *H. pylori* and reported one quasi-controlled human study in which standard quadruple therapy was combined with lycopene supplementation (30 mg/day). The results showed no statistically significant improvement in eradication compared with standard therapy alone after about one month. The authors stressed the urgent need for larger, blinded RCTs before any conclusions can be drawn about the role of lycopene in *H. pylori* management.

Moreover, long-term epidemiological studies also provide little support for lycopene as a protective factor against gastric cancer. Han et al. [147] found no significant association between dietary lycopene and gastric cancer risk, with odds ratios and relative risks hovering near null. Although cancer risk reduction differs from acute gastroprotection, this finding underscores that dietary lycopene alone is unlikely to be a dominant preventive factor at the population level.

Despite the growing body of evidence supporting the biological activities of lycopene, several mechanistic aspects remain incompletely understood. Although numerous studies have identified the involvement of signalling pathways such as Nrf2, NF-κB, PI3K/Akt, AMPK, and SIRT1, the precise molecular targets of lycopene have not yet been fully characterized, particularly in human subjects. Furthermore, it remains unclear whether the reported biological effects are mediated primarily by lycopene itself or, at least in part, by its metabolites, including apo-lycopenoids generated through oxidative or enzymatic cleavage processes. Differences in metabolism, bioavailability, and tissue distribution may further contribute to the variability observed among experimental and clinical studies. Therefore, additional mechanistic investigations and well-controlled human intervention studies are required to better define the molecular basis of lycopene activity and its physiological relevance [43].

## 7. Future Challenges and Directions

Although there are several studies that detail the benefits of dietary lycopene, there are still limitations when it comes to lycopene’s poor solubility and stability. Hence, to overcome them, innovative formulations must be taken into consideration. One of those formulations is in the form of self-emulsifying drug delivery systems (SEDDS). A lycopene formulation using red guava oil showed enhanced stability and antioxidant effects in keratinocytes [148]. Developed for wound healing, lipid liquid crystal nanoparticles (LLCs) increased lycopene’s solubility and potential therapeutic efficacy [149]. Nanomicelles and nanocarriers have been shown to promote the nano-delivery of lycopene with an improved bioavailability. Recent literature describes how nanoencapsulation, polymeric nanocarriers, and polyelectrolyte complex nanoparticles significantly enhance lycopene’s solubility, protect it from degradation (light, heat, oxidation), and improve its absorption in vivo [28,120]. Recent studies demonstrated that nanostructured lipid carriers (NLCs) formulations can enhance lycopene’s solubility, stability, and bioaccessibility, while PLGA or PLGA–PEG nanoparticles provide controlled and sustained release, supporting targeted delivery and improved therapeutic potential [28,150].

In terms of future randomized controlled trials should explore more the combinations lycopene–polyphenols combinations in several conditions Integration of genomic, metabolomic, and microbiome data could enable tailored nutraceutical approaches, aligning with the personalized nutrition paradigm.

## 8. Conclusions

Lycopene is especially notable due to its chemical structure, its high capacity to neutralize reactive oxygen species and that it may be involved in the modulation of lipid metabolism, endothelial function, and inflammatory responses, which could be relevant in the context of cardiovascular and metabolic disease prevention. Unlike previous reviews, the present work offers an integrated and application-oriented perspective on the role of lycopene in tomatoes, combining aspects of phytochemistry, bioavailability and nutraceutical relevance with a regional analysis of dietary supplements, while highlighting both recent advances in its characterization, extraction and bioavailability, as well as nutraceutical implications and current limitations in clinical validation. In recent years, modern extraction techniques such as ultrasound-, microwave-, enzyme-assisted, and supercritical fluid extraction have been increasingly explored, with generally promising results in terms of efficiency and reproducibility. At the same time, advanced analytical methods, including HPLC and mass spectrometry, remain essential for the accurate characterization and quality assessment of such extracts. Encapsulation approaches and nanostructured delivery systems have also attracted attention, as they can improve the stability and solubility of lycopene, and, in many cases, its bioavailability. Compared to dietary sources, lycopene supplements provide a more controlled and standardized intake, whereas tomato-based products may benefit from the combined action of multiple bioactive compounds present in the natural matrix. Both forms appear to be generally well tolerated within the studied dosage ranges, although their role in the prevention of specific diseases is not yet fully established and requires further clinical validation. Overall, additional long-term clinical studies and pharmacokinetic investigations are needed to better clarify the effects of lycopene, as well as its interactions with other bioactive compounds. Future research should also continue to focus on optimizing the extraction methods, improving its phytochemical characterization, and evaluating the extract stability, in order to support the development of effective biomedical and nutraceutical applications.

## Figures and Tables

**Table 1:** Lycopene content in various food matrices.

Matrix	Lycopene Content (mg/100 g FW)	Source (Author, Year)
Fresh tomato (raw)	2.5–8.0	[45,46]
Sun-dried tomato	45.9	[5]
Tomato paste	5.4–150	[47]
Ketchup	11.6–25.5	[48]
Pink guava	5.23–5.50	[49]
Watermelon	2.30–7.20	[49]
Pumpkin	1.4–7.4	[50]
Papaya	0.11–5.30	[9]
Gac fruit	19–50	[51]
Rosehip	0.68–0.71	[49]
Pink grapefruit	1.1–3.4	[47]

**Table 2:** Extraction methods and analytical techniques used for lycopene recovery from tomato-based matrices reported in the literature.

Source	Extraction Method	Solvent	Extraction Conditions	Analytical Technique	Key Parameters	Lycopene Concentration	References
Tomato pomace spouted bed-dried	MAE	EtOH/EA (90:10, *v*/*v*)	T = 90 °C; t = 3 min; S/L = 1:20 (*w*/*v*)	HPLC-UV	C18 column MeOH/ACN (90:10) 0.8 mL/min λ = 470 nm	59.66 µg/g extract	[62]
Tomato pomace spouted bed-dried	PLE	EtOH/EA (50:50, *v*/*v*)	T = 90 °C; Flow = 2 mL/min; P = 100 bar; S/L = 1:20 (*w*/*v*)			20.09 µg/g extract	
Tomato skin powder	IL-MAE	1-butyl-3-methylimidazolium chloride	P = 800 W; t = 150 s; S/L = 15:1 mL/g	UV-Vis	λ = 470 nm 5000 rpm, 5 min	0.157 mg/g DW	[63]
Dried Biomass (DB)	UAE	Ethanol	T = 65 °C, t = 20 min, L/S = 72 mL/g, A = 65%, on = 33 s, V = 90 mL	HPLC	C18 RP column ACN/MeOH/IPA (44:54:2, *v*/*v*/*v*) 1 mL/min λ = 470 nm R^2^ = 0.982	1536 µg/g DB	[64]
Tomato skins powder	UAE	n-hexane	T = 36 °C, t = 20 min, L/S = 20 mL/g, A = 70%,	HPLC-DAD	C18 column MeOH/CAN (9:1) + 0.125% TEA 1.2 mL/min λ = 475 nm R^2^ = 1	735.9 mg/g DE	[66]
		α-pinene				468.1 mg/g DE	
		VNADES Menthol:thymol (1:1)				484.2 mg/g DE	
		VNADES Menthol:thymol (1:1) (azeotropic mixture)				358.7 mg/g DE	
Industrial grated tomato by-products (freeze-dried weight)	Pulsed UAE	Ethanol	T = 45 °C, t = 28 min, S/L = 2 g/25 mL, A = 82%,	UHPLC-PDA	C32 column λ = 476 nm R^2^ = 0.797	2586 mg/kg FDW	[67]
Tomato peels (dried powder)	UAE–MAE (synergistic)	Ethyl lactate	T = 45 °C, t = 8 min, S/L = 0.03 g/mL, MW = 500 W, US = 600 W (pulse 1 s on/1 s off)	HPLC-DAD	YMC C30 column	37.08 mg/100 g peels	[68]
Tomato peels (dried)	Soxhlet extraction	Hexane/Acetone (1:1, *v*/*v*)	t = 4 h, 10 siphon cycles, S/L = 0.05 g/mL, solvent evaporation at 40–45 °C			40.66 mg/100 g peels	
Tomato seeds (powder)	Conventional stirring	Hexane	4 h, RT, S/L = 0.1 g/mL			19.66% oil yield	
	Soxhlet extraction	Food-grade hexane	t = 3 h, 20 siphon cycles, S/L = 0.05 g/mL; 2500 rpm, 10 min; solvent evaporation at 38 °C			19.18% oil yield	
	UAE–MAE (synergistic)	Hexane	T = 40 °C, t = 10 min, S/L = 0.05 g/mL, MW = 500 W, US = 450 W			19.53% oil yield, 38 mg/100 g oil lycopene	
	PLE	Hexane	T = 100 °C, 1750 psi t = 6 min, S/L = 0.05 g/mL			17.17% oil yield, 44 mg/100 g oil lycopene	
Tomato waste dried powder	UAE	NADES ChCl:1,3-Butanediol (1:5)	T = 65 °C, t = 12 min, S/L = 1:20 (*w*/*v*), US power = 70%	HPLC-DAD	C18 column CAN/MeOH/DCM (7:1:2, *v*/*v*/*v*) 0.05% TEA, 0.1% BHT), 0.8 mL/min V = 20 µL λ = 473 nm R^2^ > 0.999	215.13 µg/g DW	[15]
Tomato peels dried powder	PEF pretreatment + UAE	DES Menthol:Camphor (1:1)	PEF: 6 min, 2 μs pulse, specific energy = 22.38 J/mL T = 25 °C, 640 W t = 30 min, S/L = 2 g/25 mL	HPLC-DAD	C30 column 0.8 mL/min V = 10 µL λ = 280 nm	282.55 mg/mL	[75]
Fresh tomato peels	SFE	SC-CO_2_	P = 40 MPa; T = 74 °C; t = 155 min; CO_2_ flow rate = 0.32 kg/min; fractional separation: S45 (5 MPa, 25 °C)	UV–Vis (470, 503 nm); HPLC-DAD (450 nm)	RP-18 column, CAN 90%/Ethyl Acetate	39.11 mg/g DW	[69]
Tomato slices + 20% seed powder	SFE	SC-CO_2_	P = 350–450 bar; T = 70 °C; CO_2_ flow = 9–13 kg/h; t = 600 min; 180 g sample (150 g TS + 30 g seeds)	UV–Vis	325–575 nm, acetone/hexane	1172.32 mg lycopene/100 g DW	[76]
Tomato pomace	EAE + FEP (Pectinase + Cellulase + Fast Extraction)	Buffer acetat pH 4.7 + Ethyl acetate	T = 30 °C; t = 30 h (enzymatic); enzymes: 0.48 mL/g (P) + 0.42 mL/g (C); shake 4 g; FEP 40 mL Ethyl acetate	HPLC	C30 column 1.3 mL/min λ = 475 nm	1568 µg lycopene/g DW	[71]
Tomato peel fraction						3216 µg lycopene/g DW	
Dried tomato skins	EAE + UAE	n-hexane	m = 0.1 g; V = 5 mL; T = 25–45 °C; t = 5–120 min; Enzymatic treatment = 60–180 min (0.1 mL Lallzyme EX-V + 2 mL H_2_O, 25 °C)	UV–Vis spectrophotometry	λ = 502 nm	1120 mg/kg DW	[72]
Dried tomato peed	EAE (pectinase) + UAE	Distilled water (pH 7)	2 g sample + 20 mL water; pH = 7; 55 °C; 24 h (enzymatic); 100 µL pectinase; ultrasonication 24 W, 28 kHz, 30–60 min	UV–Vis spectrophotometry	λ = 503 nm	5.22 mg lycopene/100 g DW	[77]

**Table 3:** Encapsulation methods used for lycopene.

Method	Matrix Composition	Technique	Size	Lycopene Content	Stability	Encapsulation Efficiency (EE%)	Advantages	Use	References
Polymeric nanoparticles	PLGA + PVA (polylactic-co-glycolic acid + polyvinyl alcohol)	Double emulsion solvent diffusion	393.5 nm	3.2 µg/mg	Good stability up to 5 months at 4 °C	Not reported	Improved antioxidant stability	Skin delivery	[79]
Polymeric nanoparticles	PLGA + Tween 20	Double emulsion solvent diffusion	177.6 nm	2.1 µg/mg	Fairly stable after 2 months at 4 °C	Not reported	Smaller particle size improves penetration	Skin delivery	[79]
Polymeric	PLGA	Ultrasonication emulsification	103 nm	Not reported	Improved thermal stability	87	Controlled release and improved bioavailability	Nutraceutical delivery	[78]
Polyelectrolyte complex nanoparticles	TLH-3 polysaccharide + sodium caseinate	Electrostatic complexation	241 nm	10.03%	Improved thermal and light stability	93.6	Improved water dispersibility and antioxidant activity	Nutraceutical/pharmaceutical delivery	[80]
Chitosan-alginate nanocapsules	Chitosan + sodium alginate	Gelation	148.7–152.8 nm	Not reported	Improved antioxidant stability	86.85	Increased bioavailability	Nutraceutical systems	[81]
Niosomes	Span 60 + cholesterol	Microfluidic preparation	237–281 nm	Not reported	Thermal stability	>90	Uniform size distribution	Topical delivery	[82]
Niosomes	Span + Cholesterol	Thin-film hydration	245–457 nm	Not reported	Thermal stability	>90	Controlled release	Topical delivery	[82]
Spray-dried microcapsules	Maltodextrin (coating agent) + nanoemulsion (isopropyl myristate + Pluronic F-127)	O/W nanoemulsion preparation (rotor-stator + ultrasonication) followed by spray drying	259–276 nm	Not reported	~50% degradation after 1 month	99	Formation of stable powder Improved dispersion	Food/nutraceutical ingredient	[84]
Self-emulsifying delivery system (SEDS)	MCT (medium-chain triglycerides)/sunflower oil/oleic acid + Tween 80 + Span 80	Low-energy self-emulsification	181.70–572.27 nm	up to 29.87 ± 0.16 mg/g	stable W/O/W emulsions	Not reported	Improved solubility and bioaccessibility	Functional foods/nutraceutical delivery	[83]

**Table 4:** Representative lycopene supplements available in the United States, Europe, and Romania.

Product	Formulation Type	Declared Lycopene per Unit	Recommended Daily Dose	Source of Lycopene	Country	References
Lyc-O-Mato Lycopene (Swanson)	Softgels	10 mg	1 softgel/day	Lyc-O-Mato^®^ tomato extract	USA	[101]
Lycopene 10 mg (NOW Foods)	Softgels	10 mg	1 softgel/day	Tomato extract	USA	[102]
Natural Lycopene (Healthy Origins)	Softgels	15 mg	2 softgels/day	Lyc-O-Mato^®^ tomato extract	USA	[103]
Mega Lycopene (Life Extension)	Softgels	15 mg	1 softgel/day	Tomato extract	USA	[104]
Lycopene 20 mg (NOW Foods)	Softgels	20 mg	1 softgel 1–2 x/day	Lyc-O-Mato^®^ tomato extract	USA	[105]
GNC Lycopene 30 mg	Softgels	30 mg	2 softgels/day	Lyc-O-Mato^®^ tomato extract	USA	[106]
Puritan’s Pride Lycopene 40 mg	Softgels	40 mg	1 softgel/day	Tomato extract	USA	[107]
Nutricost Lycopene 50 mg	Softgels	50 mg	1 softgel/day	Tomato extract	USA	[108]
Lycopene 10 mg (Jamieson)	Tablets	10 mg	1 tablet/day	Tomato concentrate	Canada	[109]
Ultra Lycopene (Vitabiotics)	Tablets	15 mg	1–2 tablets/day	Tomato extract	UK	[110]
Lycopene (GymBeam)	Capsules	10 mg	1 capsule/day	Tomato extract	Slovakia	[111]
Lycopene Lyc-O-Mato 15 mg (Doctor Life)	Capsules	15 mg	1 capsule/day	Lyc-O-Mato^®^ tomato extract	Poland	[112]
Licopen 15 mg (Rotta Natura)	Capsules	15 mg	1–2 capsules/day	Tomato extract	Romania	[113]
Licopen (Favisan)	Capsules	15 mg	1–2 capsules/day	Tomato extract + tomato powder	Romania	[114]
Phyto Licopen (Pro Natura/Medica)	Capsules	60 mg	1–3 capsules	Tomato powder, lycopene powder	Romania	[115]

## Data Availability

No new data were created or analyzed in this study. Data sharing is not applicable.
